# Supplementary material for: Comparative genomics provides new insights into the diversity, physiology, and sexuality of the only industrially exploited tremellomycete: Phaffia rhodozyma
Source: BMC Genomics. 2016 Nov 9;17:901. doi: 10.1186/s12864-016-3244-7 (PMC5103461; doi:10.1186/s12864-016-3244-7)
Supplement: Additional file 6: — List of orphan genes with links to PFAM (related to Additional file 1: Table S1). (ZIP 1428 kb) [file 12864_2016_3244_MOESM6_ESM.zip › PFAM_UNIQUE/G05542_P.html]

#                                                                            --- full sequence --- -------------- this domain -------------   hmm coord   ali coord   env coord
  
# target name        accession   tlen query name           accession   qlen   E-value  score  bias   #  of  c-Evalue  i-Evalue  score  bias  from    to  from    to  from    to  acc description of target
  
#------------------- ---------- ----- -------------------- ---------- ----- --------- ------ ----- --- --- --------- --------- ------ ----- ----- ----- ----- ----- ----- ----- ---- ---------------------
  
G05542\_P             -            348 SSP160               PF06933.6    756       7.4    3.1   7.9   1   2       6.7   1.4e+03   -4.5   0.4   317   334   125   142   120   145 0.70 - Special lobe-specific silk protein SSP160  
G05542\_P             -            348 SSP160               PF06933.6    756       7.4    3.1   7.9   2   2    0.0013      0.27    7.8   2.1   298   339   247   288   238   314 0.85 - Special lobe-specific silk protein SSP160
